# Intra-operative ultrasound in the surgical treatment of complex and recurrent pilonidal disease: a retrospective, observational, single-center study

**DOI:** 10.1007/s00384-025-04961-3

**Published:** 2025-09-20

**Authors:** Gaetano Gallo, Marta Goglia, Veronica De Simone, Gianpiero Gravante, Pierpaolo Sileri, Antonio Crucitti, Marco La Torre

**Affiliations:** 1 Department of Surgery, Sapienza University of Rome, Rome, Italy; 2 Department of Medical and Surgical Sciences and Translational Medicine, Faculty of Medicine and Psychology, Sapienza University of Rome, Rome, Italy; 3 Department of General Surgery, Azienda Sanitaria Locale ASL Lecce, Casarano, Italy; 4 Colorectal Surgery Unit, IRCCS San Raffaele Scientific Institute, Vita-Salute University, 20132 Milan, Italy; 5 Department of Surgery, Ospedale Cristo Re, Rome, Italy

**Keywords:** Intra-operative ultrasound, Pilonidal disease, Single-center study

## Abstract

**Background:**

Pilonidal disease (PD) is frequently associated with high recurrence rates and delayed healing, particularly in complex or recurrent cases. While Endoscopic Pilonidal Sinus Treatment (EPSiT) has improved postoperative recovery and patient satisfaction, its effectiveness can be limited by incomplete identification of fistulous tracts. Intraoperative ultrasound (IUS) offers real-time visualization of subcutaneous structures and may aid in detecting hidden tracts during surgery. This study evaluates the clinical outcomes of combining IUS with EPSiT in the treatment of complex and recurrent PD.

**Materials and methods:**

A retrospective cohort, single-center study was conducted on patients with recurrent and complex PD treated between 2018 and 2021 using IUS in conjunction with EPSiT. All patients had a minimum follow-up of 36 months. The study recorded the number of cases in which IUS identified additional fistulous tracts and led to a modification of the surgical strategy, as well as clinical outcomes including recurrence rate, time to wound healing, and incidence of incomplete wound healing.

**Results:**

Nineteen patients were included (14 males, 73.7%; mean age of 35.4 ± 6.4 years). The mean operative time was 42 min, with IUS requiring an additional 6 min. IUS identified previously undetected fistulous tracts in 6 patients (31.5%), leading to modifications in the surgical strategy. At 36-month follow-up, disease persistence (recurrence or incomplete healing) was observed in 5 patients (26.3%). Recurrent cases were successfully managed with additional procedures, achieving 100% healing after reintervention.

**Conclusions:**

Intraoperative IUS identified previously undetected secondary tracts in 31.5% of patients, leading to a modification of the surgical approach. Further comparative studies are needed to validate its effectiveness and assess its potential role as a standard adjunct in the surgical management of pilonidal disease.

## Introduction

Pilonidal disease (PD) primarily affects young adults and often leads to discomfort, reduced quality of life, and frequent interruptions in work or school activities. While conventional surgical treatments remain in use, the past decade has seen a growing interest in minimally invasive techniques (MITs) that aim to reduce tissue trauma, shorten recovery times, and improve aesthetic and functional outcomes [1–3]. Various MITs have been developed with these goals in mind, including pit-picking, laser ablation, phenol injection, fibrin glue instillation, endoscopic pilonidal sinus treatment (EPSiT) and video-assisted ablation of the pilonidal sinus (VAAPS) [4]. These procedures share several advantages — limited surgical access, preservation of surrounding tissues, reduced postoperative pain, faster return to daily activities, and good cosmetic results. However, differences in technique, case selection, and outcome reporting still limit their direct comparability, and long-term recurrence data are often lacking.

Among the MITs, EPSiT has gained increasing popularity since its introduction by Meinero et al. in 2013 [5]. EPSiT allows direct visualization and removal of the sinus tracts using a fistuloscope, enabling targeted treatment while minimizing skin incisions and tissue disruption. The technique has been associated with healing rates of up to 95%, rapid postoperative recovery (often within 4 weeks), low complication rates, and excellent patient satisfaction [6, 7]. In addition, EPSiT is performed under local or regional anesthesia, often as an outpatient procedure, which further enhances its appeal in the management of PD [6, 7].

Despite these encouraging results, recurrence may still occur, often due to incomplete removal of secondary tracts or unrecognized extensions of the disease [8]. To overcome this limitation, intraoperative ultrasound (IUS) is being explored as a complementary tool to improve the accuracy of tract identification during MITs. By enabling real-time visualization of hidden or secondary pathways, IUS may help refine the endoscopic approach, potentially reducing recurrence and improving outcomes. The aim of the present study is to evaluate the surgical outcomes of combining IUS with EPSiT in the management of complex and recurrent PD. In particular, the study aims to describe the role of IUS in identifying additional fistulous tracts and its impact on modifying the surgical strategy, as well as to report clinical outcomes such as recurrence rate, healing time, and incomplete wound healing.

## Materials and methods

This a retrospective cohort, observational, single-center study. It has been reported according to the Strengthening the Reporting of Observational Studies in Epidemiology (STROBE) guideline [9].

**Fig. 1:**
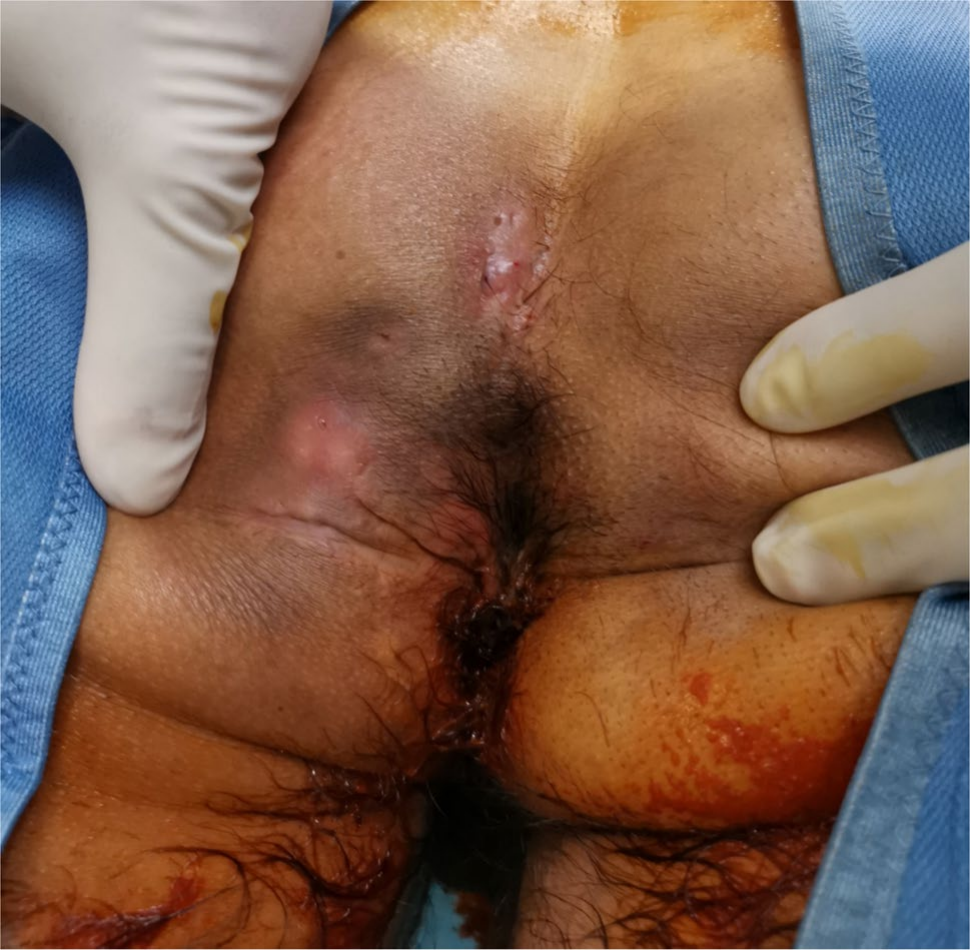
A 42-year-old male patients with complex pilonidal disease

All patients treated between January 2018 and August 2021 for recurrent and complex PD who had reached a minimum follow-up of 36 months were retrospectively analyzed. Complex disease was defined as the presence of at least three external orifices (including both midline and off-midline openings) in association with at least two subcutaneous fistulous tracts (Fig. 1). Patients with less severe disease or with follow-up shorter than 36 months were excluded. Patient selection was performed using a prospectively maintained institutional database. Informed consent was obtained from all individual participants included in the study.

All patients underwent EPSiT combined with sinusectomy, performed in a day surgery setting under local anesthesia (10 ml of 2% mepivacaine hydrochloride and 10 ml of 7.5% ropivacaine hydrochloride) and superficial sedation. Prior to the surgical procedure, all patients underwent intraoperative ultrasound (IUS) using the Esaote MyLab Sigma system with an L4-15 ZIF linear array probe (5–13 MHz). The entire sacrococcygeal region was systematically scanned to identify any secondary tracts or abscesses associated with the known PD. Fistulous tracts identified with IUS corresponded to those not detectable during intraoperative inspection and digital palpation. All additional tracts or abscesses detected on IUS were marked on the skin and addressed during surgery either through the EPSiT technique or via sinusectomy/sinusotomy, depending on the anatomical findings. In this context, sinusectomy referred to the surgical excision of the entire sinus tract with minimal surrounding tissue, typically reserved for fibrotic or branched disease. Sinusotomy was defined as the deroofing of the sinus tract to allow healing by secondary intention, without excising surrounding tissue. The choice between the two approaches was based on intraoperative anatomical assessment and tract complexity.

### Outcomes of the study

The primary outcomes of the study were the number of cases in which IUS identified additional fistulous tracts not detectable by visual inspection or digital palpation, and the number of cases in which these findings resulted in a modification of the planned surgical strategy. Secondary outcomes included operative time (including the additional time required for IUS), time to complete wound healing, incidence of incomplete wound healing, recurrence rate, and number of days off work. Wound healing time was calculated from the day of the surgical procedure to the date on which the surgical wound was considered completely healed during clinical follow-up. A wound was defined as completely healed when full epithelialization was observed, with no signs of discharge, inflammation, pain, or open sinus tracts on inspection and palpation. This assessment was performed by the operating surgeon during scheduled outpatient visits at the 1 st postoperative week and month, and subsequently at 6, 12, and 36 months after surgery. For the purpose of this study, incomplete healing was defined as the failure of the surgical wound to fully close or epithelialize within 36 months of follow-up. Recurrence was defined as the return of symptoms or the development of new fistulous tracts after an initial period of complete healing. The persistence rate was calculated as the sum of cases with incomplete healing and those with recurrence over the 36-month follow-up. All cases of incomplete healing or recurrence were followed until complete resolution was achieved through additional treatments.

### Statistical analysis

All data were inserted into an Excel database (Microsoft, Redmond, Washington – United States) and analyzed with the Statistical Package for the Social Sciences Windows version 27.0 (SPSS, Chicago, Illinois, USA). Descriptive statistics used were the mean ± standard deviation for continuous parametric variables, the median and interquartile range for continuous non-parametric variables and frequencies for categorical variables. Normality assumptions were demonstrated with histograms and the Kolmogorov/Smirnov test.

## Results

Nineteen patients with recurrent or complex PD were enrolled in the study. The cohort included 14 males (73.7%), with a mean age of 35.4 ± 6.4 years. Four patients (21.1%) were obese, and two (10.5%) were smokers. Seven patients (36.8%) had recurrent disease, with recurrences occurring on average 13.7 ± 7.6 months after the initial surgery. Initial surgery consisted in a closed technique (*n* = 4, 57.1%), open technique (*n* = 1, 14.3%), EPSiT (*n* = 1, 14.3%), and pit picking (*n* = 1, 14.3%). The mean number of openings was 3.6 ± 1.2. In nine patients (47.4%), these openings were located along the midline (“pits”), while in the remaining 10 patients (52.6%) they were found laterally (“secondary lateral openings”).

**Fig. 2:**
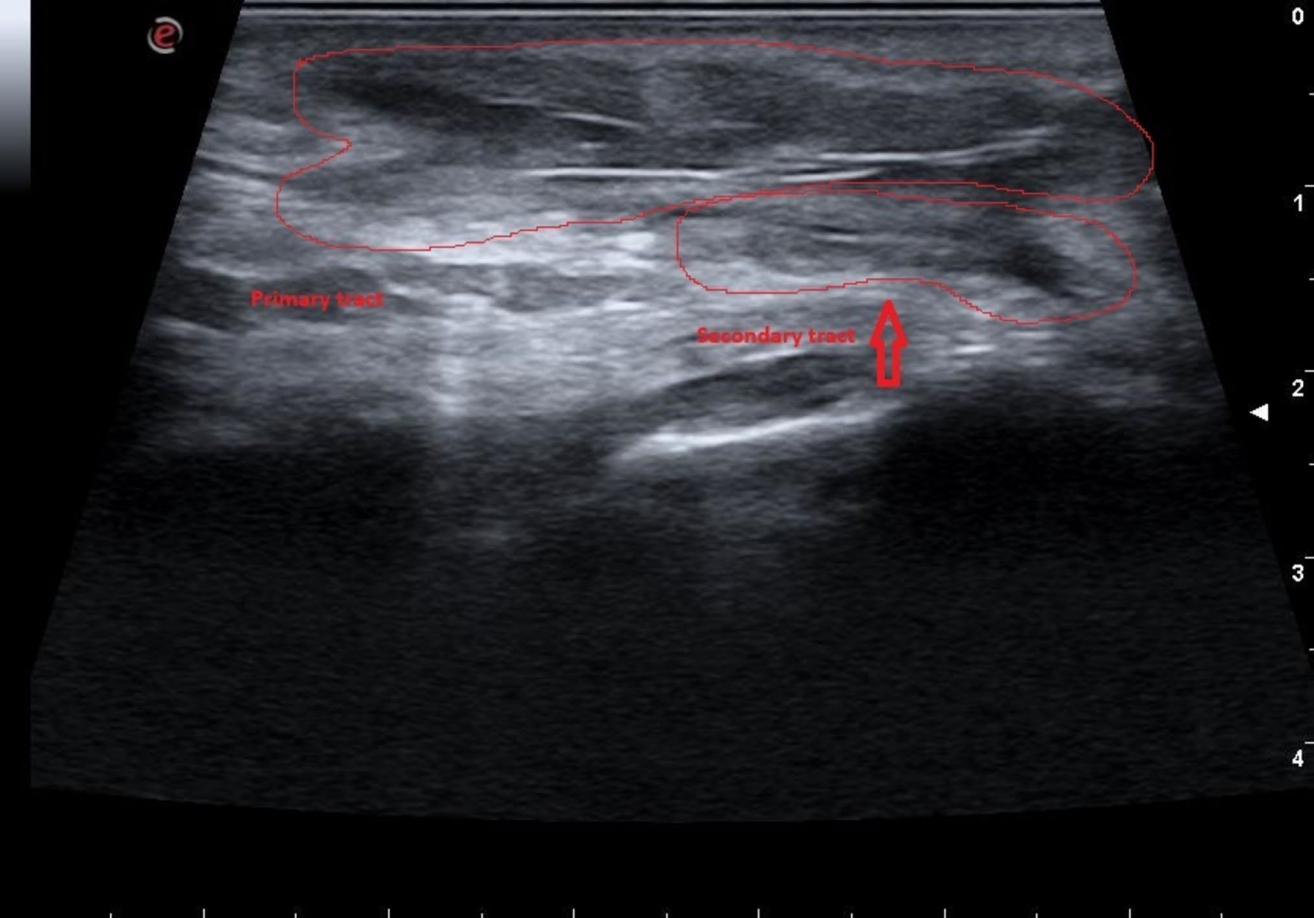
Ultrasonographic identification of both primary and secondary (deeper and potentially unrecognizable without the use of IUS) fistulous tracts

**Fig. 3:**
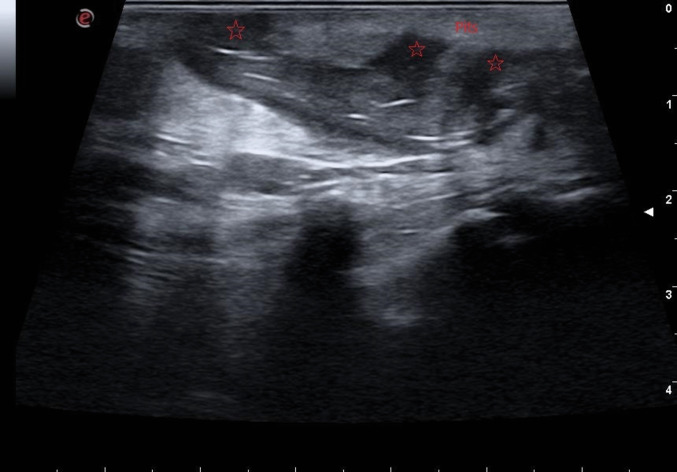
Star-shaped figures indicate multiple pits

**Fig. 4:**
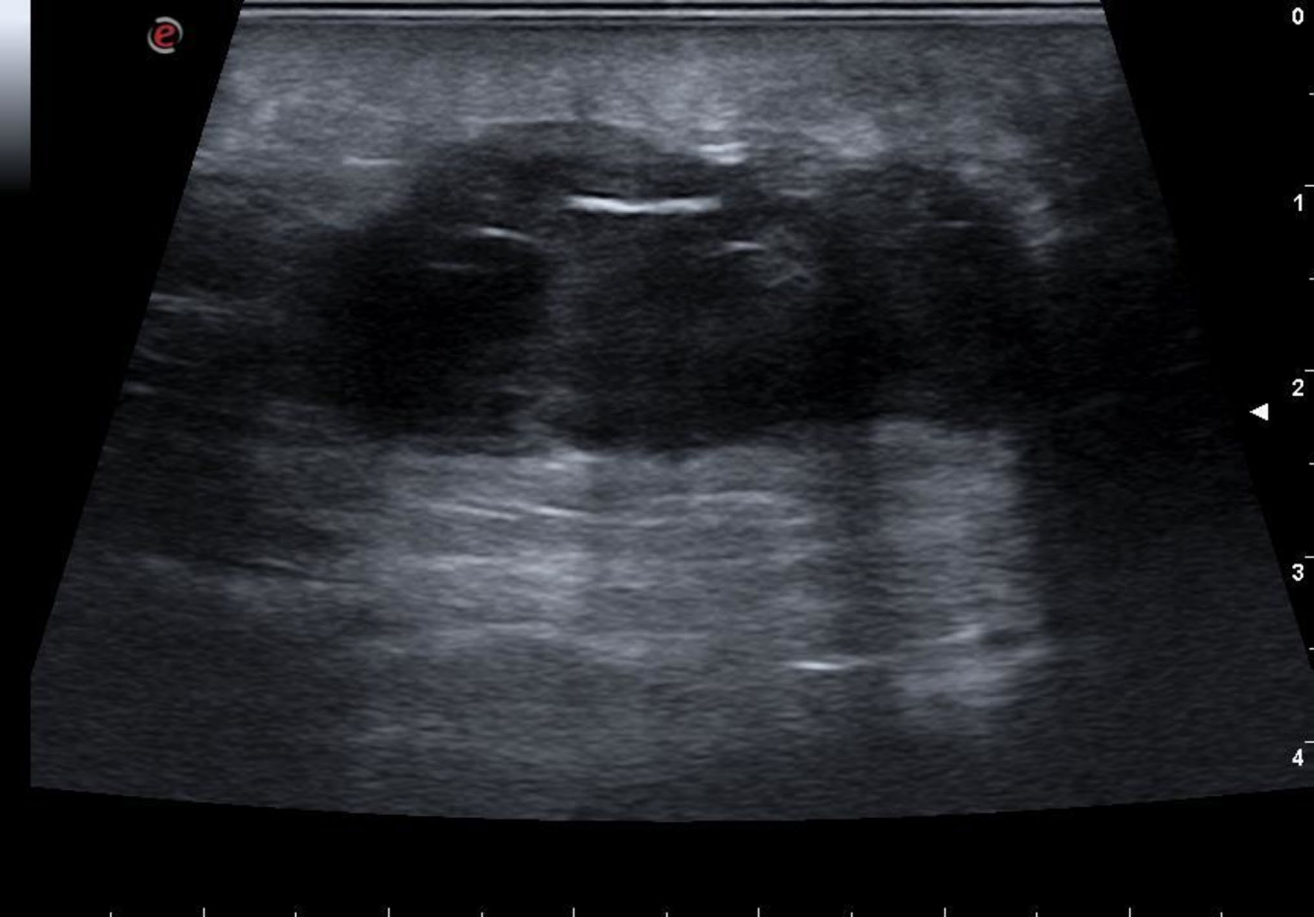
Secondary gluteal abscess

**Fig. 5:**
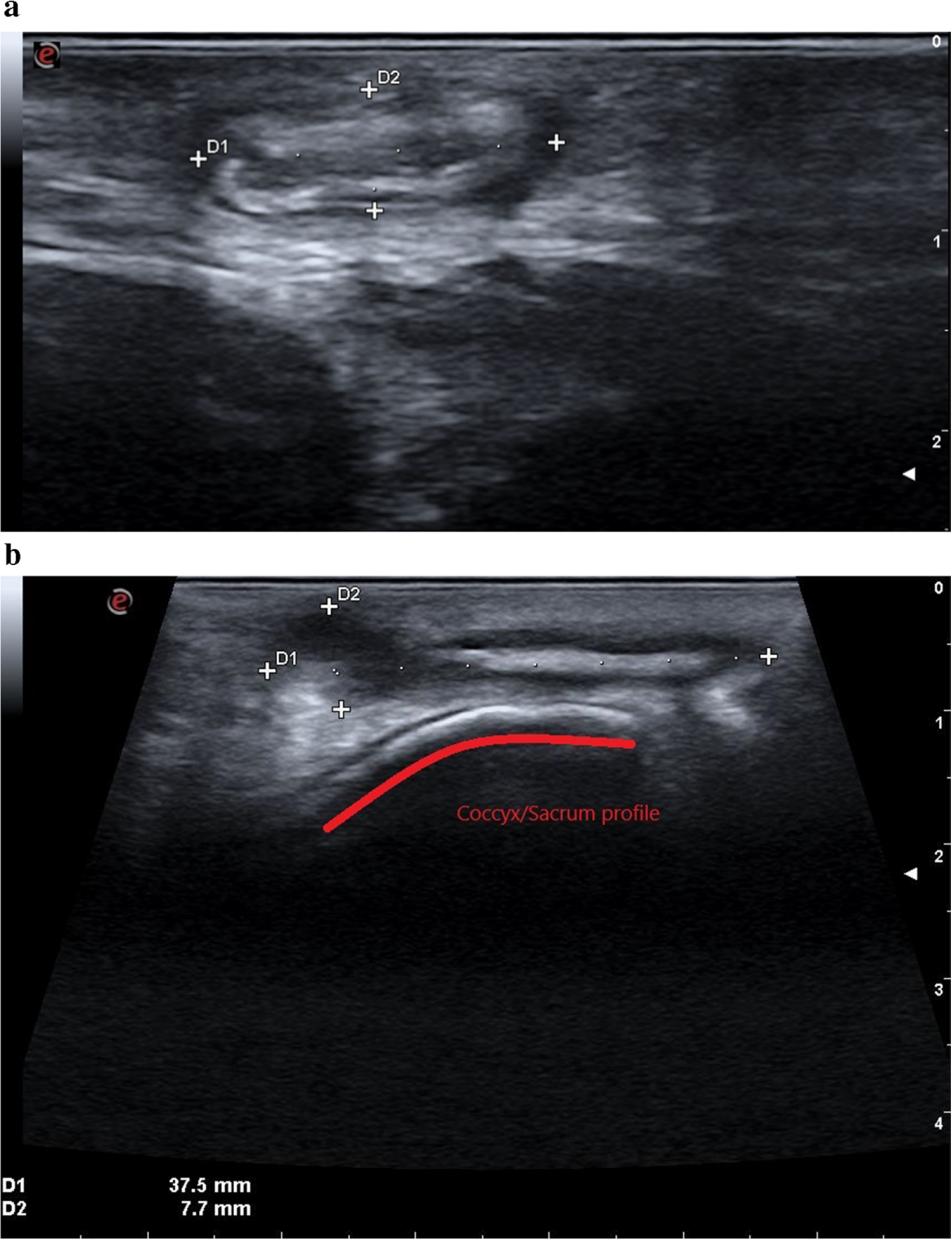
**a** Demarcation of the fistulous tract and **b** identification of the sacrum/coccyx profile

The mean operative time for the surgical procedure was 42 min (range 35–55), with IUS assessment requiring an additional 6 min on average (range 4–9). In six patients (31.5%), IUS identified one or more fistulous tracts that had not been detected during the initial exploration (Figs. 2, 3, 4, 5). In all cases, an additional sinusectomy was performed alongside the planned EPSiT procedure to allow for complete removal of the fistulous components.

The average time to wound healing was 46.95 ± 21.46 days, with a median of 1.26 ± 0.871 day off work (range 1–6). At the 36-month follow-up, two cases of recurrence (10.5%) and three cases of incomplete wound healing (15.8%) were observed. When combined, these outcomes resulted in an overall persistence rate of 26.3%. Among the five patients with persistent disease, two underwent redo-EPSiT, one underwent sinusotomy, and two underwent sinusectomy as second-line treatments. Three of these patients (60%) achieved complete healing and were considered cured. The remaining two patients required a third surgical procedure (sinusectomy), which resulted in complete wound healing in both cases. Reoperations achieved complete resolution of persistent disease in all patients within 6 months.

## Discussion

In the present series, IUS identified previously undetected fistulous tracts in 31.5% of cases, enabling a more accurate assessment of PD anatomy and prompting changes in the surgical strategy. This underscores the potential of IUS to enhance intraoperative decision-making, particularly in complex and recurrent cases where standard visual inspection may be insufficient. Our findings align with existing literature. In a recent retrospective analysis, repeated IUS assessments during the pit-picking procedure revealed that 61% of patients had asymptomatic residual or newly formed sinuses up to 12 mm in size [10]. These subclinical elements − often invisible to direct inspection − can significantly impact recurrence rates if not identified and treated. Furthermore, as demonstrated by Manigrasso et al., recurrence is closely related to disease complexity, including the presence of undiagnosed lateral tracts, residual hair, or small niduses of infection, and is exacerbated by the lack of a standardized classification system [8]. Incorporating IUS into the routine surgical evaluation of PD may therefore help overcome these limitations, offering a more thorough anatomical mapping and potentially reducing the risk of incomplete treatment.

In terms of feasibility, the use of IUS proved to be both straightforward and efficient, with an average duration of just 6 min per procedure. This minimal time investment did not compromise the quality of the surgical intervention; rather, it enhanced intraoperative precision by enabling the identification of secondary fistulous tracts and any associated abscesses directly by the operating colorectal surgeon [11, 12]. From an economic perspective, IUS did not introduce additional costs. The necessary equipment — typically consisting of a linear vascular probe and a standard ultrasound platform − is often already available in most surgical settings. This makes the technique an economically sustainable and widely adoptable adjunct to EPSiT and other MITs. MRI has shown usefulness in assessing the extent of pilonidal disease and detecting secondary tracts, but its limited availability and high costs represent significant drawbacks [13].

Finally, IUS plays a pivotal role in supporting the implementation of MITs, as the precise mapping of the disease enables targeted treatment of affected tissues. For instance, Papagiannopoulos et al. demonstrated that real-time IUS-guided laser ablation of PD and its associated tracts was both safe and effective [14]. This approach minimizes the risk of damaging healthy subcutaneous tissue, reduces the likelihood of treatment failure, and ensures more complete tract ablation. Consequently, integrating IUS with MITs may result in shorter recovery times, reduced tissue trauma, and improved postoperative quality of life by limiting scarring and enhancing cosmetic outcomes.

Our findings should be interpreted in light of several limitations. First, although this study highlights the potential utility of IUS in guiding surgical management of complex or recurrent PD, it did not account for key patient-related covariables such as smoking status, obesity, or other established risk factors for recurrence. The absence of such data limits the ability to fully assess their potential confounding effect on surgical outcomes. Second, the retrospective design and the relatively small sample size, drawn from a single tertiary referral center, limit the generalizability of our results. Given the potential clinical impact of integrating IUS into EPSiT protocols, future research should consider a matched case–control design comparing patients treated with and without IUS, ideally in a larger cohort, including a systematic analysis of comorbidities and other factors predictive or recurrence. This would enhance the statistical validity and allow a more nuanced evaluation of outcomes.

## Conclusions

The adoption of IUS in support of the surgical treatment of PD seems to offer several benefits, including a low recurrence rate, ease of use, ability to identify hidden fistula tract, minimal additional time required, cost-effectiveness, and support for MIT. Further research is warranted to fully establish its efficacy and potential as a standard component of PD surgery (Tables 1, 2, 3).

**Table 1:** Demographic variables

Population	
F/M	5/14 (26.3%/73.7%)
Age (years)	35.4 ± 6.379
Obese population (n°)	4 (21.1%)*
Smoking population (n°)	2 (10.5%)

*BMI > 30

**Table 2:** PD characteristics and pits location. * n° (%)

Mean number of pits	3.58 ± 1.216	
	n. 7	1 (5.3%)
	n. 5	2 (10.5%)
	n. 4	6 (31.6%)
	n. 3	7 (36.8%)
	n. 2	3 (15.8%)
Lateral location*	10 (52.6%)	
Medial location*	9 (47.4%)	
Recurrent sinus*	7 (36.8%)	
	*Type of previous surgery*	
	Closed technique*	4 (57.1%)
	Open technique*	1 (14.3%)
	EPSiT*	1 (14.3%)
	Pit picking*	1 (14.3%)
Months from previous surgery	13.7 ± 7.521

**Table 3:** Procedural results. * n° (%)

Time to wound healing (days)	46.95 ± 21.46	
Time off work (days)	1.26 ± 0.871	
Incomplete wound healing*	3 (15.8%)	
Recurrence at 24 months*	2 (10.5%)	
Persistence at 24 months*	5 (26.3%)	
First operation in all patients	EPSiT + sinusectomy	Healing rate 14/19 (73.7%)
Second operation in case of recurrence (5 cases)	2 EPSiT 2 sinusectomy 1 sinusotomy	Healing rate 3/5 (60%)
Third operation in case of re-recurrence (2 cases)	2 sinusectomy	Healing rate 2/2 (100%)

## Data Availability

No datasets were generated or analysed during the current study.
